# Supplementary material for: Topologically distinct 2D and 3D intratumoral heterogeneity scores for preoperatively predicting invasiveness in stage I lung adenocarcinoma: A multicenter study
Source: PLOS Digit Health. 2026 Feb 20;5(2):e0001246. doi: 10.1371/journal.pdig.0001246 (PMC12923145; doi:10.1371/journal.pdig.0001246)
Supplement: S1 Appendix — Detailed specifications of the CT acquisition protocols used at the three participating medical centers. (PDF) [file pdig.0001246.s001.pdf]

S1 Appendix: CT Acquisition Protocols

Preoperative thoracic CT examinations were conducted across multiple institutions using multidetector computed tomography systems. The Affiliated Hospital of Southwest Medical University utilized uCT 550 or CT 760 scanners (Shanghai United Healthcare), whereas The Third Xiangya Hospital of Central South University and Xiangtan Central Hospital employed Brilliance iCT (Philips Healthcare, Netherlands) and Revolution 256 CT (GE Healthcare, USA), respectively.

The imaging procedure involved helical scanning in a supine position, with patients instructed to hold their breath while images were captured from the upper lung to below the costophrenic angle. The parameters for volumetric CT imaging included a tube voltage of 120 kV, a tube current-time product ranging from 180 mA to 280 mA, and a matrix size of 512×512. Initial image processing steps were completed before reconstruction, which was conducted using a standard algorithm. The slice thickness and intervals varied between 0.55 mm and 0.625 mm.
