# Supplementary material for: Topologically distinct 2D and 3D intratumoral heterogeneity scores for preoperatively predicting invasiveness in stage I lung adenocarcinoma: A multicenter study
Source: PLOS Digit Health. 2026 Feb 20;5(2):e0001246. doi: 10.1371/journal.pdig.0001246 (PMC12923145; doi:10.1371/journal.pdig.0001246)
Supplement: Attachment — Submitted filename: Response to Reviewers.pdf [file pdig.0001246.s003.pdf]

Response to Reviewers

January 13, 2026

Manuscript Information

Manuscript ID: PDIG-D-25-00799

Manuscript Title: Topologically Distinct 2D and 3D Intratumoral Heterogeneity Scores for Preoperative Prediction of Invasiveness in Stage I Lung Adenocarcinoma: A Multi-center Study

Dear Editor and Reviewers,

We would like to thank you for your insightful comments and constructive suggestions regarding our manuscript (**PDIG-D-25-00799**). We have carefully revised the manuscript in accordance with the reviewers' comments. A point-by-point response to each comment is provided below. All changes in the revised manuscript have been highlighted.

Journal Requirements

REQ 1.

Comment: *Please provide an Author Summary. This should appear in your manuscript between the Abstract and the Introduction, and should be 150–200 words long.*

Response: Thank you for this instruction. We have added the following Author Summary between the Abstract and Introduction as requested:

Author Summary: Lung adenocarcinoma is the predominant form of lung cancer, and for early-stage patients, surgical decisions hinge on accurately predicting tumor invasiveness. Distinguishing between non-invasive lesions suitable for limited resection and invasive tumors requiring lobectomy remains challenging with standard subjective CT interpretation. To address this, we developed a quantitative framework that analyzes the internal "texture" and structural complexity of lung nodules. A key innovation of our study is the introduction of a "3D Intratumoral Heterogeneity Score," which uses advanced topological analysis to map the spatial connectivity and fragmentation of tumor tissue across the entire volume, rather than just a single 2D slice. We integrated these scores into a machine learning model and validated its performance on a large cohort of 1,238 patients from three different medical centers. Our results confirm that this 3D approach significantly outperforms traditional methods in identifying invasive cancer. This non-invasive, robust tool offers clinicians a powerful objective metric to guide personalized surgical planning, helping to avoid overtreatment and preserve vital lung function for patients.

REQ 2.

> **Comment:** *If the reviewer comments include a recommendation to cite specific previously published works, please review and evaluate these publications...*

Response: We have carefully reviewed all suggested references and incorporated relevant citations into the revised manuscript to strengthen our discussion and context.

Additional Editor Comments

ED 1.

> **Comment:** *Provide more detail on the radiomics feature extraction process.*

Response: We have significantly expanded **Section 3.3** ("Calculation of Topologically Distinct 2D/3D ITH Scores") and updated Algorithm 1 to provide a granular description of the feature extraction workflow. Specifically, we have clarified that:

- For the **2D ITH score**, local radiomic features are extracted using a 2×2 sliding window on the largest cross-sectional slice to map planar texture patterns.
- For the **3D ITH score**, we implemented a voxel-wise feature extraction using a $2 \times 2 \times 2$ sliding window across the entire tumor volume.
- The methodology now explicitly describes the use of a **26-connectivity topology** (encompassing face, edge, and corner adjacency) to identify connected voxel components, ensuring that the metric captures anisotropic texture variations and volumetric fragmentation along the z-axis.

These revisions ensure that the voxel-based, topology-aware quantification strategy is reproducible.

ED 2.

> **Comment:** *Clarify how the optimal number of clusters (6 for 2D, 5 for 3D) was determined. Justify these choices with references or validation.*

Response: We apologize for the confusion. In our revised manuscript, we have standardized and justified the cluster number setting ($K = 6$) for consistency across both 2D and 3D modalities, based on preliminary experiments and maximizing the silhouette coefficient. This is now clarified in **Section 3.3** and Algorithm 1.

ED 3.

> **Comment:** *Explain the handling of missing data and class imbalance in model training.*

Response: We appreciate the opportunity to clarify these points:

• **Missing Data:** We would like to confirm that **there were no missing values** in the final dataset used for model development. This was ensured by our strict enrollment criteria and rigorous data cleaning process, where complete clinicoradiologic documentation was a mandatory prerequisite for patient inclusion.
• **Class Imbalance:** To address the uneven distribution of pathological subtypes, we employed two strategies: (1) **Stratified sampling** was strictly applied during the train-test split to maintain consistent class ratios across cohorts; (2) During model training, we utilized the built-in **class-weighting mechanisms** of the ensemble algorithms (e.g., CatBoost's `auto_class_weights` parameter) to automatically adjust the penalty for misclassifying minority samples, ensuring balanced sensitivity and specificity.

ED 4.

> **Comment:** *In Table 1, correct the typo in AIC/MIA to AIS/MIA.?*

Response: Thank you for pointing this out. We have corrected the typo "AIC/MIA" to "AIS/MIA" in Table 1.

ED 5.

> **Comment:** *Ensure all figures (especially Figs. 4–8) are clearly labeled and legible in the final version.*

Response: We have replaced Figures 4–8 with high-resolution versions. Axis labels, legends, and text annotations have been enlarged for better readability.

ED 6.

> **Comment:** *Discuss potential overfitting given the high performance in external validation.*

Response: We have added a discussion regarding the high external validation performance. While uncommon, we attribute this to the rigorous feature selection process (SHAP-based elimination) and the robustness of the 3D ITH score. We also discuss cohort composition factors that might have contributed to this result.

ED 7.

> **Comment:** *Abstract: Improve the abstracts readability; ensure it clearly states the clinical impact.*

Response: We have rewritten the Abstract to improve flow and clearly highlight the clinical implications of using topologically distinct ITH scores for surgical decision-making.

ED 8. **Comment:** *Language: Check grammar and syntax throughout, especially in the Introduction and Discussion.*

Response: The entire manuscript has been thoroughly proofread and edited by a native English speaker to ensure grammatical accuracy and clarity.

Response to Reviewer #1

R1.1. **Comment:** *The introduction should more clearly articulate the novelty of the 3D ITH score compared to existing 2D methods and habitat imaging.*

Response: We have significantly revised the Introduction to emphasize the concept of "topologically distinct" subregions. We explicitly explain how the proposed 3D ITH score captures volumetric connectivity and spatial complexity, which are missed by planar (2D) analysis and traditional habitat imaging.

R1.2. **Comment:** *A direct comparison with prior studies (e.g., Li et al., 2023) would help contextualize the contribution.*

Response: We have included a discussion comparing our method with Li et al. (2023) and other relevant studies, highlighting our contribution in introducing voxel-based topological connectivity.

R1.3. **Comment:** *The use of different CT scanners and protocols across centers may introduce variability. Were any image harmonization techniques applied?*

Response: We acknowledge that scanner variability is a common challenge in multicenter radiomics studies. In this work, specific image harmonization techniques (e.g., ComBat) were not applied to the raw images. However, our methodology is designed to be inherently robust to such variations.

As detailed in **Section 3.3**, **"To quantify the internal heterogeneity of LUAD nodules, we developed a dual-scale computational framework that transforms local radiomic features into topologically distinct tissue subregions."** This approach extends established methodologies [18-23] by implementing a **voxel-based topology-aware quantification strategy**.

The key advantage of this strategy is that it relies on the **spatial arrangement and connectivity** of tumor subregions (topology) rather than absolute radiomic feature values. The unsupervised clustering step adapts to the intensity distribution of each individual scan, effectively normalizing inter-scanner differences. Previous multicenter studies utilizing similar ITH frameworks [18-23] have demonstrated that such topology-based metrics maintain high robustness across different centers. Our results further validate this, as the model achieved stable and even superior performance in the external validation set (AUC=0.881) despite the use of different CT systems.

R1.4.

> **Comment:** *The manuscript should provide a clear specification of the optimization criterion used for hyperparameter tuning (AUC or ACC).*

Response: We have clarified in **Section 3.5.1** that the Area Under the Curve (AUC) from out-of-fold (OOF) validation was used as the primary optimization criterion for hyperparameter tuning.

R1.5.

> **Comment:** *The methodological description of the SHAP-based ablation experiment requires greater clarity.*

Response: We have revised **Section 3.5.3** and the caption of Algorithm 2 to clearly outline the stepwise sequence: calculation of global SHAP importance, ranking, sequential forward selection, and identification of the "knee point" (ϵ threshold).

R1.6.

> **Comment:** *Equation 3 should be reformulated to explicitly indicate that the optimization process is initiated after computing the absolute SHAP values...*

Response: We agree that the logical flow from SHAP value computation to feature optimization needed to be more explicit. We have revised **Section 3.5.3** ("Model Interpretability and Incremental Feature Optimization") to clearly distinguish between the calculation of individual SHAP values and the subsequent ranking metric used for optimization.
Specifically, immediately following Equation 3 (which defines the SHAP value ϕ_i), we have explicitly formulated the **Global Importance Score (I_i)** to demonstrate that the optimization is based on the mean absolute magnitude:

$$I_i = \frac{1}{N} \sum_{j=1}^{N} |\phi_i^{(j)}|$$

We further clarified the procedural sequence in the text: **"For each model, the constituent features were ranked in descending order based on their Global Importance Score (I_i)... Based on these rankings, we executed a sequential forward selection process..." [Section 3.5.3]**. This ensures the reader understands that the optimization process (Algorithm 2) is strictly initiated *after* the computation and aggregation of absolute SHAP values.

R1.7. **Comment:** *The Discussion should expand on its potential clinical utility (feasibility, cost-effectiveness, barriers).*

Response: We appreciate this suggestion. We have expanded the **"Limitations"** section in the Discussion to explicitly address the translational aspects of our model.
Specifically, we added the following text: **"Clinically, this approach is highly cost-effective as it utilizes standard preoperative CT images without additional expense. However, routine implementation faces barriers, specifically the need for software integration into radiological workflows and prospective standardization of diagnostic cut-offs."**
This addition provides a balanced view of the method's feasibility and the challenges remaining before routine implementation.

R1.8. **Comment:** *The Discussion should delve deeper into the factors contributing to the superior performance of the CatBoost classifier.*

Response: We have added a paragraph in the Discussion analyzing CatBoost's strengths, particularly its effective handling of features and robustness against overfitting, which made it suitable for our dataset.

R1.9. **Comment:** *To improve model interpretability, the inclusion of a SHAP beeswarm plot is recommended.*

Response: We have updated Figure 7 to provide a more comprehensive visualization of feature importance. We believe the current representation (bar chart + detailed importance analysis) clearly conveys the dominance of the 3D ITH score.

R1.10. **Comment:** *The study lacks histopathological correlation of the ITH subregions. This should be highlighted as a key future direction.*

Response: We agree. We have added this to the "Limitations" section, suggesting that future studies should aim to correlate imaging habitats with spatial transcriptomics or multi-region pathology.

Response to Reviewer #2

R2.1. **Comment:** *The first highlighted contribution does not sufficiently explain the advantages of the proposed 3D ITH.*

Response: We have revised the Introduction and the Highlights to explicitly state that the 3D ITH score quantifies "volumetric heterogeneity by integrating local and global patterns," capturing anisotropic variations missed by 2D methods.

R2.2. **Comment:** *An important relevant reference, "Multiperspective tumor heterogeneity metrics...", is missing.*

Response: We have cited this relevant work (Ref [23] in the revised manuscript) and discussed its relevance in the Introduction.

R2.3. **Comment:** *In Section 2.2, if the radiologists are authors, explicit indication is recommended.*

Response: The radiologists involved in segmentation are authors of this paper. We have clarified their roles in the "CRediT authorship contribution statement".

R2.4. **Comment:** *In Section 2.3, the sentence ending with using volumetric CT images: should conclude with a period.*

Response: We appreciate the reviewer's careful reading. The punctuation at the end of the specified sentence in **Section 3.3** has been corrected to a period.

R2.5. **Comment:** *On page 3, the last line preceding Equation 1 contains only a single 1, which looks visually unbalanced.*

Response: We have adjusted the typesetting to ensure Equation 1 is displayed correctly and the surrounding text flows naturally.

R2.6. **Comment:** *In Section 2.3.1, use metric or measure instead of calculations in the subheading.*

Response: We have renamed the subheading to "2D ITH Score Derivation" (and similarly for 3D) to be more accurate (now in **Section 3.3.1**).

R2.7. **Comment:** *The authors are encouraged to release the core code.*

Response: We have included a "Data Availability" statement indicating that data and code are available upon reasonable request.

R2.8. **Comment:** *The image clarity appears to be insufficient. The font size in Figure 2 and Figure 6 appears too large.*

Response: We have uploaded higher-resolution versions of all figures and adjusted the font sizes in Figures 2 and 6 to be consistent with the rest of the manuscript.

R2.9. **Comment:** *There seems to be a blank image at the end of Figure 3. The bottom part of Figure 4 appears to be truncated.*

Response: We have corrected Figure 3 to remove the blank space and fixed the truncation issue in Figure 4.

R2.10. **Comment:** *The total number of references seems relatively small.*

Response: We have added more recent and relevant references to the Introduction and Discussion sections to support our findings.

R2.11. **Comment:** *The captions of the subfigures are currently placed above the images. It would be more appropriate to place them below.*

Response: We have adjusted the layout so that all subfigure captions and main figure legends are placed below the images, adhering to standard formatting.

R2.12. **Comment:** *The Conclusion section could be more comprehensive. Please consider adding a description of the methodological innovations.*

Response: We have rewritten the Conclusion to explicitly mention the "volumetric ITH quantification" and "topologically distinct" aspects of our methodology.

Response to Reviewer #3

R3.1. **Comment:** *The robustness and methodological justification of the 3D ITH metric require further clarification, specifically regarding cluster number selection (K).*

Response: We have clarified in the revised method section that we consistently employed $K = 6$ clusters for both 2D and 3D scores to ensure comparability. This choice was based on optimization experiments (silhouette analysis) described in the Methods. We believe this consistency strengthens the comparative analysis.

R3.2. **Comment:** *The model achieved slightly better performance metrics in the external validation cohort than in the internal test set. This merits further clarification.*

Response: We have added a paragraph in the Discussion addressing this observation. We believe it may be due to the specific composition of the external cohort (e.g., clearer distinction in features for that specific population) and demonstrates the strong generalizability of the 3D ITH score. We have discussed potential contributing factors.

R3.3. **Comment:** *Figure 7 (rose diagram) lacks clear axes and scales.*

Response: We have revised the caption for Figure 7 to clearly explain the metrics represented. The visualization aims to show the relative importance of features, with the 3D ITH score dominating the importance ranking.
