# Supplementary material for: Topologically distinct 2D and 3D intratumoral heterogeneity scores for preoperatively predicting invasiveness in stage I lung adenocarcinoma: A multicenter study
Source: PLOS Digit Health. 2026 Feb 20;5(2):e0001246. doi: 10.1371/journal.pdig.0001246 (PMC12923145; doi:10.1371/journal.pdig.0001246)
Supplement: Attachment — Submitted filename: comment-PDIG-D-25-00799.docx [file pdig.0001246.s002.docx]

The 3D ITH employed in this study is a novel approach for quantifying tumor heterogeneity and has been validated on a large-scale multi-center dataset. I recommend that the manuscript be accepted after major revision.

1. The first highlighted contribution does not sufficiently explain the advantages of the proposed 3D ITH. Please further elaborate on the benefits of employing 3D ITH.

2. An important relevant reference, Multiperspective tumor heterogeneity metrics for preoperative prediction of IASLC grading in clinical stage IA lung adenocarcinomas: A multicenter study, is missing. Please include a brief discussion of this work in the Introduction.

3. As mentioned in the first comment, the current description of the advantages of the proposed 3D ITH in the final part of the Introduction is not sufficiently detailed. The authors are encouraged to provide a more comprehensive explanation of its strengths.

4. In Section 2.2, a radiologist with 5 years of experience and another with 10 years of experience are mentioned. If these radiologists are among the authors of the paper, it is recommended to explicitly indicate their names.

5. In Section 2.3, the sentence ending with ‘using volumetric CT images:’ should conclude with a period.

6. On page 3, the last line preceding Equation 1 contains only a single‘1’, which looks visually unbalanced. Please adjust the typesetting accordingly.

7. In Section 2.3.1, the subheading ’2D ITH score calculations’ seems slightly inappropriate. It may be more accurate to use ‘metric’ or ‘measure’ instead of ‘calculations’.

8. Making the code publicly available would greatly help other researchers reproduce the results and support further development in the community. The authors are encouraged to release the core code.

9. The image clarity appears to be insufficient. Please provide higher-resolution versions of the figures.

10. The font size in Figure 2 and Figure 6 appears too large. Please reduce it appropriately.

11. There seems to be a blank image at the end of Figure 3. Does it have any specific meaning? If not, it can be removed.

12. The bottom part of Figure 4 appears to be truncated. Please provide the complete image.

13. The total number of references seems relatively small. Please consider adding more references to demonstrate that the study is based on a comprehensive literature review.

14. The captions of the subfigures are currently placed above the images. It would be more appropriate to place them below the images

15. The Conclusion section could be more comprehensive. Please consider adding a description of the methodological innovations presented in this study.
